# Health Financing Reforms in Uganda: Dispelling the Fears and Misconceptions Related to Introduction of a National Health Insurance Scheme: Comment on "Health Coverage and Financial Protection in Uganda: A Political Economy Perspective"

**DOI:** 10.34172/ijhpm.2022.7348

**Published:** 2022-07-03

**Authors:** Robert K. Basaza, Prossy K. Namyalo, Boniface Mutatina

**Affiliations:** ^1^School of Medicine, Uganda Christian University, Mukono, Uganda.; ^2^School of Public Health, Gudie University Project, Kampala, Uganda.; ^3^Institution of Health Policy, Management and Evaluation, University of Toronto, Toronto, ON, Canada.; ^4^Faculty of Social Sciences, Ndejje University, Kampala, Uganda.; ^5^College of Health Sciences, Makerere University, Kampala, Uganda.

**Keywords:** Reforms, Political-Economic Analysis, User Fees, Health Insurance, Uganda

## Abstract

Uganda introduced health financing reforms that entailed abolition of user fees, and in due process planned to introduce a National Health Insurance Scheme (NHIS). This paper accentuates a contextual and political-economic analysis that dispels the fears and misconceptions related to introduction of the insurance scheme. The Grindle and Thomas model is used to depict how various factors affect decision making by policy elites concerning a particular policy at a particular time. Drawing lessons from the sub-Sahara region and in particular, Ghana and Rwanda’s experience, it is clear that the political will of the executive led by the president in many countries is a key determinant in bringing about health reforms. In this paper, we provide insights based on contextual and political-economic analysis to countries in similar setting that are interested in setting up NHISs.

## Introduction

 This paper provides a contextual and political-economic analysis of health financing reforms in Uganda and in particular dispelling the fears and misconceptions related to introduction of a National Health Insurance Scheme (NHIS). It presents national and regional evidence with regard to the upcoming Ugandan scheme. Since 1986 when the current government took power, it embarked on enacting health reforms to enhance public health system financing. Meanwhile, the country was emerging out of a 5-year protracted guerilla war as such the healthcare system was fragile and needed revitalization. Among the issues of concern were irregular salary payment of health workers, shortages of medicines and lack of community engagement in health facilities’ management. During this period, the World Bank and International Monetary Fund were promoting user fees as an alternative health financing mechanism that would reduce government expenditure and increased borrowing.^1^ However, in 2000s, it was evident that user fees were insufficient in raising significant revenue for health facilities and they were found to reduce the poor people’s demand and access to health services.^1^

 Amidst the user fees failure debate in 2001, Uganda publically abolished user fees, in favor of providing free healthcare services in public healthcare facilities. Political commentators considered user fees removal as a political pledge fulfillment by the current President during the 2000 election campaigns. During these campaigns, the electorates had decried the high costs of healthcare services which left many of them without any option but foregoing the needed services.^2-4^ Although this reform did not result in zero cost to households since patients had to incur related costs to healthcare like transport, meals accruing from the need to eat due to long hours spent at the facility. The reform triggered a surge in demand for especially, outpatient care services which were noted to have more than doubled.^3^ Moreover, benefits of user fees removal in Uganda especially to the lower quintile population have been documented to include universal coverage of expanded immunization program and antenatal clinics, increased use of the lower-level government health facilities, and per capital outpatient attendance. Notably, it improved access to health services among the poorest households.^3^ Over the years, this policy has drifted as evidenced by the increasing out of pocket health expenditures.^4^ Despite this, the current government has not introduced any new reforms to reduce on the financial hardship being faced by Ugandans. Accordingly, this has always been perceived to mean that the current government – president and the ruling party – are not interested in major policy reforms that contradict the current free healthcare policy. This paper first embarks on dispelling fears and misconceptions related to the political elites.^5^

## Analysis of the Process

 In this analysis, the Grindle and Thomas model was used to demonstrate how various factors affect decision making by political elites concerning a particular policy at a given time. The role of political elites in population policy although not emphasized in scholarship, actually rests in systematic understanding of their part during any policy change or reform. Thomas and colleague simply put it, only when political elites notice an issue as a crisis is when they will make it appear on the agenda. This paper depicts that Ugandans’ thinking is in analogue with Thomas’ scholarship. Political authorities not only posse moral rights to generate and employ positive law but the political will of their leaders can eventually influence policy changes.^6-8^

## Results and Discussion

 Drawing from the region, it is clear that the political will of political leaders in many countries is key in bringing about big health reforms. In Ghana for example, the President showed high-level political commitment and as such succeeded in having the national health insurance (NHI) law passed.^6^ Additionally, it is echoed that Rwanda’s successful NHIS is due to the President’s directive to ensure healthcare coverage to all Rwandans.^6^ The incumbent president of Uganda has neither publically blocked the proposal to start an NHIS in Uganda nor supported it. This leaves Ugandans speculating on whether the president and the ruling party are supporting the proposed NHI. This is despite the fact that the ruling party - National Resistance Movement has reflected NHIS in its manifestos for 2011-2016 and 2016-2021.^6^ Noteworthy is that, the current president has been in power for over 36 years as well as doubling as the chairman of the ruling party. Additional fears are due to the fact that it did not appear in the 2021-2026 manifesto, at a time, when the Parliament of Uganda reviewed and approved the Bill on March 31, 2021 for signing into law. However, the President has since declined to sign it. Worse still no NHIS implementation frameworks been developed yet.^6,8^ Further the bill was passed amidst contention by the minister of health wanting to withdraw it, which points to the influence by invisible power centers.^8^

 Some commentators have argued that NHIS proposal is not perceived by the current government as a good political move given its delayed processing. Their position is backed by the fact that Uganda is the only country in East Africa that has not passed a National Health Insurance Scheme Act, yet it has the highest out-of-pocket health expenditure in the region (42%). Of concern, the public health sector continues to suffer various challenges including: limited financial resources, gross personnel challenges such as low pay and low motivation; poor infrastructure and equipment; and recurring stock outs of medications and essential supplies.^8,9^ Despite this slow progress, the country has committed to NHI at key national, regional and international levels as evidenced in its protocols, policies, frameworks and strategies. For instance, the Third National Development Plan 2020–2025 emphases establishment of NHI as one of the human capital interventions. Moreso, the NHIS is included in the long-term strategic development agenda, “the Uganda Vision 2040.”^9,10^ The Uganda government ratified to Sustainable Development Goals of which 3.8 emphasized universal health coverage (with its goals of comprehensive health services coverage and financial protection for all) and has a clear roadmap to meeting its pledge.^4^ Moreover, the champions of the NHIS policy were the Parliamentary Committee on Health and by then they were from National Resistance Movement party; the government is aware of the high out of pocket expenditure despite the free health services.^9^ Experts persuasively argue, that policy elites take up an issue according to their priority and own sense of timing; noting that sometimes the decisions are rarely urgent and they make policy decisions when the time seem propitious.^5^ As such, in circumstances of politics as usual – just like in the NHIS scenario, policy elites play an important role in selecting the moment for reforms, shape the terms of debates and normally generate agreement about the need for a policy change.

 The fears and misconceptions Uganda’s commentators hold seem to be based on Rothman’s perspective of African political elites behaving as if they are creating new public policy yet nothing changes.^5^ However, the evidence adduced indicates a change of trend. There seems a dawning of a new day concerning the NHIS in Uganda. The misconceptions and fears that exist around NHIS reforms in Uganda are thus subsequently explored.

 One of the famous issues concerning NHIS reform in Uganda is about the informal sector being hard to mobilize and collect premiums from them. The difficulties in mobilizing the informal sector to pay health insurance premiums are well documented but other countries provide a learning ground on this issue.^6^ Countries like Rwanda and Ghana built their national schemes on the informal sector that is why their enrollment has grown over a time.^6^

 Ugandans cannot afford premiums and the informal sector is not willing to pay for health insurance. Commentators advance this idea building on fact that people are getting free healthcare services ignoring the available evidence, which shows vice versa. The high out-of-pocket health expenditures implies that majorities are actually paying for their healthcare services already and the country is experiencing high catastrophic expenditure that stands at 14.2%.^4^ Moreover, the community health insurance schemes have survived for almost three decades amidst provision of free healthcare services and without a supportive legal framework^6^ Recent evidence demonstrates that some members of those scheme pay up to Ugandan Shilling (UGX) 72 215/= (US$ 20.6) annually for the limited package provided by the community health insurance scheme.^6,8^ The proposed premium for the informal sector is UGX 100 000/= (US$ 28.6) for an extended package of services compared to what is provided in community health insurance schemes. Moreso, evidence on willingness to pay for health insurance by the informal sector has always been in the affirmative.^6,8^ Additionally, the Uganda National Household Survey 2019/2020 indicates a high willingness to pay for health insurances even in regions where the insurance schemes are non-existent. Noteworthy, 77.5% of the Ugandans (both in rural and urban settings) were considering joining health insurance according to the Uganda National Household Survey 2019/2020.^11-14^ Furthermore, Rwanda with one of thriving NHIS in Africa has higher poverty level at 31.1% compared to Uganda at 21.4% but it spends US$ 17 compare to Uganda’s US$ 6 on health expenditure per capita.^15^ This points out that the majority of Ugandans may need to set their priorities right but can actually finance healthcare services with ease. Moreover, evidence indicates that even the seemingly poor are willing to pay for health insurance as long as the quality of care is good and it’s available. The state could look into financing of the indigent.^13^

## Conclusion

 This commentary provides further insights to a political economy perspective presented by Nannini et el.^16^ The introduction of NHIS in Uganda has been slowed down by complex processes intertwined by political and context systems. The paper provides insights to low- and middle-income countries or similar set-up embarking on the path to introduction of a NHIS. The evidence adduced points out that having money does not necessarily lead to paying for health insurance. Further detailed political economic analysis will have to be done including interview of the top political brass of the ruling political system to elucidate the path to introduction of the NHIS in Uganda. The establishment NHIS is a political process; it is incorporated into the national long-term strategic agenda, and today’s limited political process may not necessarily be considered as the government’s permanent position!

## Acknowledgement

 The review of this manuscript by Emmanuel Otieno of Gudie University Project is acknowledged.

## Ethical issues

 Not applicable.

## Competing interests

 Authors declare that they have no competing interests.

## Authors’ contributions

 RKB and PKN conceived the paper. All the three authors raised the manuscript and reviewed the drafts.

## Disclaimer

 The authors declare that the views expressed in this manuscript are their own and not an official position of any institution or funder.

## Funding

 This study was funded from the authors’ meagre resources.
